# Development of Microcontroller-Based System for Background Radiation Monitoring †

**DOI:** 10.3390/s20247322

**Published:** 2020-12-20

**Authors:** Andriy Holovatyy, Vasyl Teslyuk, Natalia Kryvinska, Artem Kazarian

**Affiliations:** 1 Department of Computer Aided Design Systems, Lviv Polytechnic National University, 79013 Lviv, Ukraine; andrii.i.holovatyi@lpnu.ua; 2 Department of Automated Control Systems, Lviv Polytechnic National University, 79013 Lviv, Ukraine; vasyl.m.teslyuk@lpnu.ua (V.T.); artem.h.kazarian@lpnu.ua (A.K.); 3 Department of Information Systems, Faculty of Management, Comenius University in Bratislava, 25 82005 Bratislava, Slovakia

**Keywords:** background radiation monitoring system, Atmel AVR ATmega328 microcontroller (MC), Geiger-Mueller counter, Petri net model

## Abstract

An appearance of radiometers and dosimeters on free sale made it possible to provide better radiation safety for citizens. The effects of radiation may not appear all at once. They can manifest themselves in decades to come in future generations, in the form of cancer, genetic mutations, etc. For this reason, we have developed in this paper a microcontroller-based radiation monitoring system. The system determines an accumulated radiation dose for a certain period, as well as gives alarm signals when the rate of the equivalent dose exceeds. The high reliability of this system is ensured by a rapid response to emergency situations: excess of the allowable power of the equivalent radiation dose and the accumulator charge control. Further, we have composed a microcontroller electronic circuit for the monitoring radiation system. Additionally, an operation algorithm, as well as software for the ATmega328P microcontroller of the Arduino Uno board, have been developed.

## 1. Introduction

Radiation is dangerous due to its high damageability as well as the fact that it is not perceived by human sense organs. None of the human sense organs is able to detect the presence of the nearby radioactive source. This makes it possible to hide the information about accidents at nuclear power plants (NPPs) and their consequences. Even after the events at the Chernobyl nuclear power plant, the radioactive consequences contaminated the countries of Europe, and for several years in the USSR the devices for the determination of the level of radioactive pollution were forbidden for the civilian population.

The appearance of radiometers and dosimeters on free sale made it possible to provide better radiation safety for citizens. The effects of radiation may not appear all at once. The effects of radiation can manifest themselves in decades to come in future generations, in the form of cancer, genetic mutations, etc. It is enough to mention the consequences of the atomic bombings of the Japanese cities.

As the level of radiation in the human body increases, disorders occur that can lead to death in a matter of days or hours. Having established the increased level of radiation, it is necessary to immediately determine the source of radioactivity. In order to do this a radiometer-dosimeter is needed.

Ionizing radiation is any radiation that causes an ionizing medium. Ionizing radiation belongs to cosmic radiation, and its natural sources on Earth are radioactive substances distributed in the geosphere. Artificial sources of ionizing radiation are nuclear reactors, artificial radioactive isotopes, nuclear explosions, X-ray equipment, etc. (OSS-2000 [1] basic sanitary rules, NRBU-97 [2] norms of radioactive safety in Ukraine).

Nowadays, a significant proportion of the world’s electricity is generated by nuclear power plants. Most countries around the world attempt to reduce this share by replacing it with wind, solar stations, and others. At the same time, despite the very large investments in renewable and green energy, the process of replacing nuclear power plants is much slower than the world community expects. Therefore, in the near future we are forced to use nuclear power plants. On the other hand, we observe large-scale changes in the Earth’s climate with extreme conditions that threaten nuclear safety in many parts of the globe. In such conditions inexpensive and mobile tools for monitoring ionizing radiation of environment are necessary. Therefore, the development of devices and systems for radiation monitoring is a topical task.

## 2. Related Work

There are various scientific and technical articles which are devoted to the development of background radiation monitoring systems. In particular, in [3] the authors have developed a personal portable effective dose dosimeter with a radio frequency data transmission channel. The construction of the microcontroller X-ray detector has been developed. The sensor element of the device is implemented on the basis of a radiochromic film, Gafchromic EBT3, which changes its transparency under the influence of ionizing radiation.

The schematic diagram of the dosimeter and its electronic circuit have been designed and implemented. The developed dosimeter evaluates the change in the effective dose according to the degree of the sensory element transparency. The dosimeter has a radio frequency (RF) data transmission system. The active mode of the dosimeter operation performs only at the moments of data reading using external RF devices. The lifetime of a personal device for measuring an effective dose of ionizing radiation, due to the absence of its own power source, is limited by the maximum radiation dose and the property of the photodetector to record the optical stream that passes through the film sensor.

There is no aforementioned shortcoming in the developed microcontroller systems [4,5]. The implemented radiation monitoring system [4] is intended for monitoring of subatomic high-frequency particles in the gamma-radiation zone. The device is built on the PIC16F84 microcontroller and the SBM-19 Geiger–Mueller counter. Unlike other works, in experiments of which personal computers for processing and remote data transmission were used, in this work, the independent autonomous microcontroller system, which includes standard Internet protocols, has been developed.

The measurement data, as well as warning signals, are sent to the decision-making system through communication channels (e.g., the Internet, mobile phone, or radio amateur strap). The system is not portable and requires an Internet or mobile network for remote data transmission. The device for registration and measurement of radioactive radiation [5] was developed with the help of sensors and the transmission of the collected data is performed wirelessly. At the same time, the registration and measurement system of radiation is expensive, as well as not enough compact and mobile.

An example of a wireless mobile system for measuring gamma radiation using a commercial portable instrument (dosimeter-radiometer) GAMMA-SCOUT is given in [6]. Since the GAMMA-SCOUT device does not provide built-in wireless connectivity, an additional hardware module was developed. The prototype of the module is implemented on the MIC PIC16F887 by Microchip and RF-Telit LE70-868 transmitters. The paper presents the structure and algorithm of module work. At the same time, the system is commercial and contains closed software that limits its area of use.

In [7], a digital search dosimeter for measuring low-level gamma radiation was developed. The dosimeter uses a scintillation detector (scintillation counter) as a radiation detector (radioactive radiation) and the PIC microcontroller PIC16F876 to control the functions of the developed system. The microcontroller generates a frequency of rectangular shape with a defined pulse width for forming and regulating high voltage order of +1200 V. High voltage is required to bring the scintillation detector into operation.

An amplifier and signal amplifier were designed for further processing by the microcontroller. MK records the pulses from the output of the amplifier, programmatically treats them, and outputs the result. The software for the microcontroller is created in C using the PCWH compiler. There are no means of wireless data transmission and information about the parameters of the radioactive radiation detector used, which limits the scope of use of the developed device.

Worthy of attention is the development of devices for monitoring the radiation background from air and under water [8,9]. In particular, in [8] the development of an unmanned aeronautical complex for remote monitoring of background radiation is considered. The research was carried out using the developed aircraft in the exclusion zone of the Chernobyl Nuclear Power Plant. The descriptions of the created dosimeter and experimental data of the radiation background measurement with automatic recording of the longitude, latitude and height of the measuring point are given. As a drawback, it can be noted that the dosimeter does not belong to the class of portable measuring devices.

An example of a compact wireless radiation monitoring system in real-time in an underwater environment is given in [9]. The system is marked by a high selling price.

Accordingly, the best approaches and technical solutions among the above-described solutions for the range of tasks involved include the methods described in [4,7,8]. The research methods were based on conducting experiments that included measurements of radiation, and their processing and analysis with the help of self-developed hardware and software systems. The developed systems are closed and have a high price, which does not allow us to expand their functionality and modification to the corresponding operational needs.

Consequently, the analysis of the existing approaches and technical solutions makes it possible to state the necessity of developing a qualitatively new, inexpensive, open-source mobile microcontroller radiation background monitoring system. Such a system should be built on affordable and inexpensive components, and open source software with the ability to modify or extend its functionality in accordance with the user and application requirements.

The goal of the work is to develop and research a microcontroller based system for background radiation monitoring, which has low cost and a wide range of functional capabilities.

In order to achieve the goal, the following tasks have to be solved:

- Develop the block diagram and operation algorithm of the microcontroller system for monitoring radiation which is based on the modular principle.
- Develop the physical model of the low-cost microcontroller-based radiation monitoring system.
- Develop the embedded software of the microcontroller-based radiation monitoring system, which makes it possible to implement a wide range of functionality.

## 3. Development of Structure and Operation Algorithms of Microcontroller Based System for Background Radiation Monitoring

**Figure 1:**
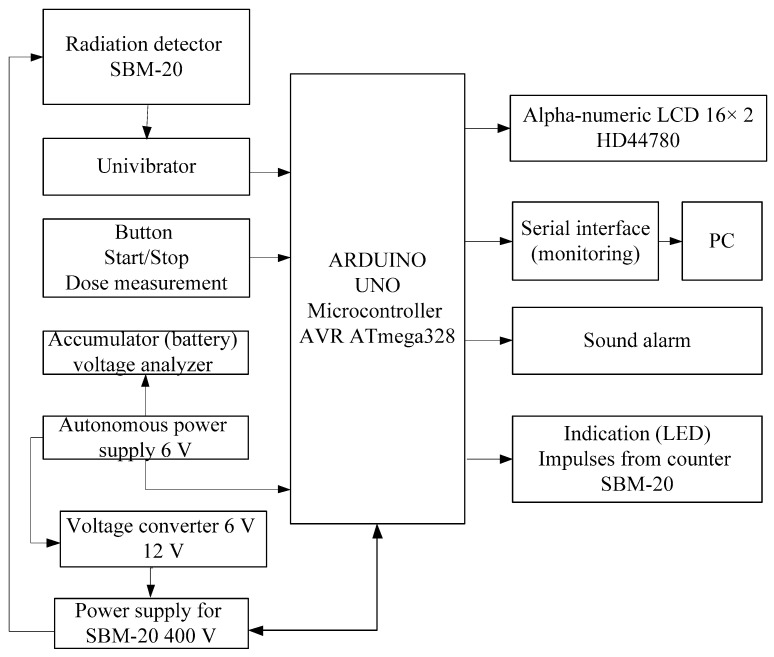
The block diagram of the microcontroller-based radiation monitoring built on the Arduino Uno board [15].

The developed microcontroller system measures the level of radiation, analyzes the received data and transmits them to the PC via serial interface. It sends the alarm signal when the level of radiation exceeds. In Figure 1, the block diagram of the microcontroller-based radiation monitoring system is shown. The system is built on the Arduino Uno board based on the ATmega328P microcontroller [10,11,12].

The developed structure includes three main subsystems, such as a subsystem for collecting the background radiation information, a data processing subsystem, and a subsystem for displaying output results. The developed structure implies the use of the modular principle in the process of the system implementation.

**Figure 2:**
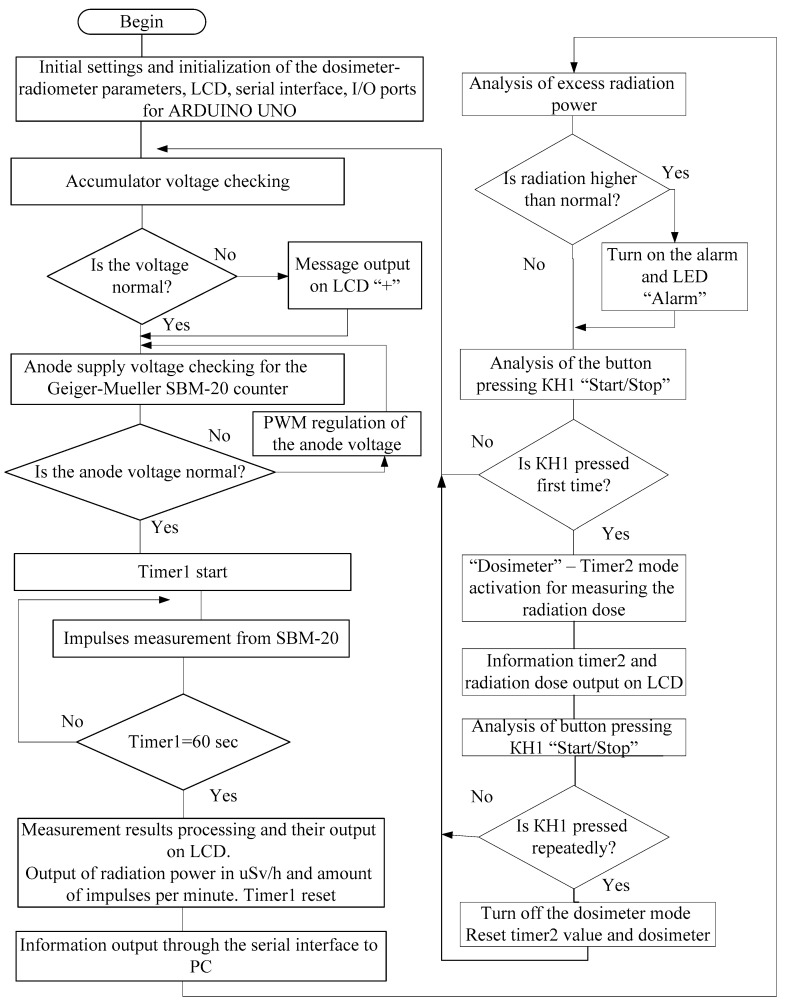
Operation algorithm of the microcontroller-based system for background radiation monitoring [15].

The algorithm of the microcontroller system operation for monitoring background radiation is shown in Figure 2. When the microcontroller software (firmware) starts, it performs its initial setup and initialization of the parameters of the dosimeter-radiometer, LCD, serial interface, and I/O ports of the Arduino Uno board [13].

In the next step, the system checks the battery voltage supply. If the voltage is normally checked for the anode voltage of the tube, the SBM-20 Geiger–Mueller counter [14] and the timer1 start. The system counts the number of impulses per 1 min with the SBM-20. Then, the system processes the measurement results and displays them on the display and via the serial interface on PC. On the liquid crystal display (LCD), the system outputs the value of the power of radioactive radiation in μSv/h and the number of impulses per minute.

Each minute the timer is reset and restarted for the next measurement. When the permissible power of radiation is exceeded, the system having a sound alarm, turns on the alarm LED. If the “Start/Stop” button is pressed for the first time, the system enters the dosimeter mode. In the dosimeter mode, timer2 starts to measure the received radiation dose. The LCD displays the irradiation dose value. If the “Start/Stop” button is pressed again, the dosimeter mode will be disabled and the system returns to the radiometer mode.

**Figure 3:**
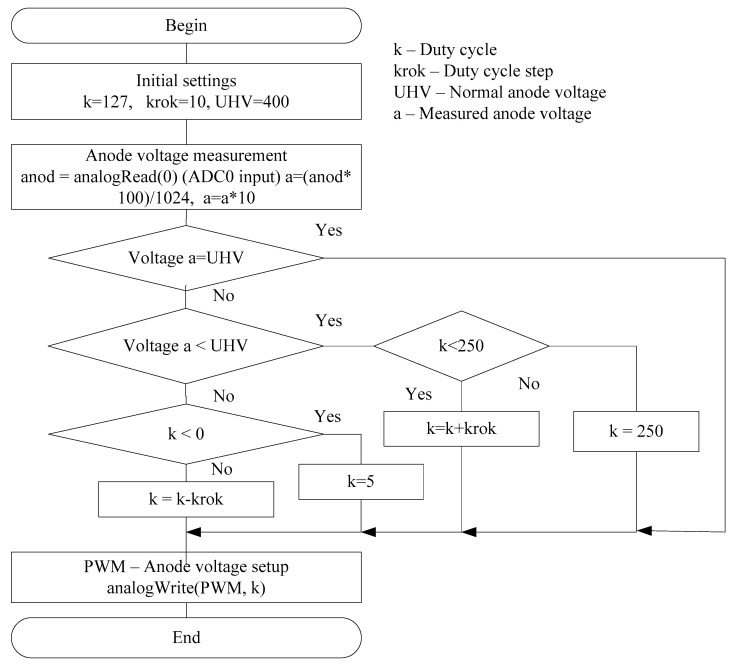
Algorithm of the anode voltage adjustment for the Geiger–Mueller counter.

The measurement accuracy depends on a number of settings for the components of the designed system. In particular, in Figure 3, we see a block diagram of the adjustment algorithm of the anode voltage for the Geiger–Mueller counter which is one of the main components of the system.

At the stage of developing the structure of the microcontroller system, the models based on theory of Petri nets have been developed [16,17,18]. The models are based on the use of the expression:

(1) $$N_{\mathrm{mod}el}=\left\{P,T,F,M_{0}\right\},P=\left\{P_{1},P_{2},\ldots ,P_{n}\right\},T=\left\{t_{1},t_{2},\ldots ,t_{m}\right\},$$

where *P* is a set of positions (states); *T* is a set of transitions; *F* is a set of arcs, which includes two subsets of input and output arcs in relation to the transition; *M*_0_ is a set of the initial marking of the Petri net; *n* is a number of positions; m is a number of conversions.

**Figure 4:**
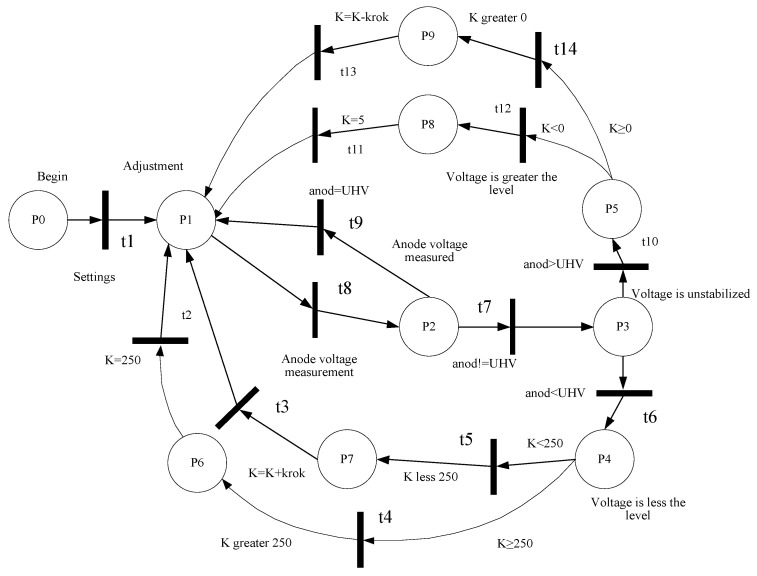
Schematic view of the model based on Petri nets for researching the process dynamics of the anode voltage adjustment for the Geiger–Mueller counter.

**Figure 5:**
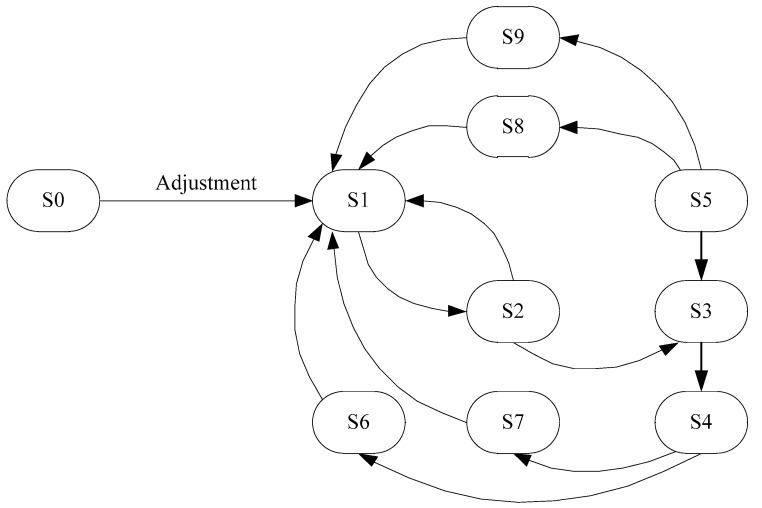
The reachability graph of the states for studying the process dynamics of the anode voltage adjustment for the Geiger–Mueller counter.

The obtained models made it possible to construct a reachability graph of states [19] in which the system may be located and to investigate the dynamics of the operation of the developed system. In particular, an example of the schematic representation of the model based on a simple Petri net for the process of the anode voltage adjustment for the Geiger–Mueller counter is shown in Figure 4. The reachability graph of states is shown in Figure 5 [20]. The developed graph allows us to obtain the information about the states in which the researched system can be:

(2) $$St=\left\{S_{0},S_{1},\ldots ,S_{l}\right\},$$

where *St* is a the set of states, and *l* is a number of states. The obtained results allow us to state that the system is alive, there are no dead ends, and the system operates correctly.

Consequently, the developed model based on the theory of Petri nets allows us to research the dynamics of the designed system.

As a result of the performed research using the developed models, based on the theory of Petri nets, it can be argued that the created Petri nets are alive, there are no dead ends, and certain states are achievable. Accordingly, it can be concluded that the designed system at the system level operates correctly and all the technical requirements are met.

## 4. Development of the Physical Model of the Microcontroller-Based System for Background Radiation Monitoring

The developed system includes hardware and software. The developed physical model of the system is shown in Figure 1. It consists of the data processing module, components of the collection of environmental data and control, and components for the processed data displaying. The data collection is implemented using a radiation detector, a gas-discharge Geiger–Mueller SBM-20 meter [14,21,22].

The control of the operating modes of the microcontroller system is performed using the key (button) (start-stop). The received data are processed by using the Arduino Uno microcontroller [10]. The processed data are displayed on the liquid-crystal display module as well as using the sound and light indication module. In addition, the physical model includes a number of modules that have been developed to ensure the functioning of the main components, such as a module for charging the battery, a DC 6 V–DC 12 V converter, a module of the anode voltage generator 400 V for power supply of SBM-20, and a univibrator circuit.

The SBM-20 counter detects beta- and gamma radiation in the range of dose rates up to 40 μR/s (0.4 μSv/h). The nominal operating voltage is 400 V. The operating voltage range is from 350 to 475 V. The voltage range is from 260 to 320 V. The range of registered capacities of exposure doses of gamma radiation is 0.004 ... 40 μR/s (0.014 ... 144 μR/h) or 0.00004 … 0.4 μSv/h (0.00014 ... 1.44 μSv/h). Sensitivity to gamma radiation from radium (226Ra) is 29 cps/mR/h (3 cps/μSv/h), and from cobalt (60Co) is 22 cps/mR/h (2 cps/μSv/h) (60 ÷ 75 cps/μR/s = 600 ÷ 750 cps/μSv/s). The plate-counting characteristic length is at least 100 V. The slope of the plate-counting characteristic is 0.1% per 1 V. Dead time at the voltage supply of 400 V is 64 R/μs. Calculation speed (counting speed) at P = 4 μR/s (0.04 μSv/s) from the source 137Cs is from 240 to 280 im/s (280 cps). The unit’s own background is no more than 1 im/s.

The dosimeter-radiometer operates on 6 V 1.2 Ah accumulator (rechargeable battery). In order to charge the accumulator (rechargeable battery), the special module is used. In order to provide the required voltage for the high-voltage converter, the 12 V DC voltage is used which is received from the DC-DC converter. The DC-DC converter converts the input voltage from the accumulator (5.5–6.8 V rechargeable battery) to 12 V. The DC-DC converter is built with an MC34063 chip, The MC34063 chip operates in the circuit as the step-up converter.

The transistor Q1 is used to increase the power of the converter to 3 A. Using the potentiometer RV1 the output voltage can be adjusted. In order to operate the Geiger counters need a high voltage source. Usually, such sources are autonomous. One of the classic converters is a 12 V to 400 V converter. 

The circuit of the pulse-width modulator (PWM oscillator) is built using a 555 timer IC. With the potentiometer RV1 the oscillator frequency is adjusted, and with the potentiometer RV2, the oscillator duty cycle. The impulses of the required frequency and duty cycle arrive at the gate of the transistor Q1. The transistor Q1 increases the voltage on the coil L1 to 400 V. The accurate voltage value is adjusted with the potentiometer RV2. From the capacitor C4, the high voltage is applied to the Geiger counter (tube). The frequency of the oscillator impulses is 4–14 kHz. The high voltage oscillator can be implemented on the microcontroller which provides PWM functions. From the output PE3 (OC3A) to the input Q1 the PWM sequence arrives that sets the voltage to 400 V.

For adjusting the output voltage in the required range we apply the divider R6-RV3 to the microcontroller ADC input ADC0. Depending on the voltage value at the output PE3 (OC3A) the necessary PWM sequence is generated.

The Arduino Uno board based on the ATmega328P microcontroller is the main module for processing data from the Geiger–Mueller counter (tube), displaying the needed information on the LCD module, sending data to the PC through the serial port [15,23].

**Figure 6:**
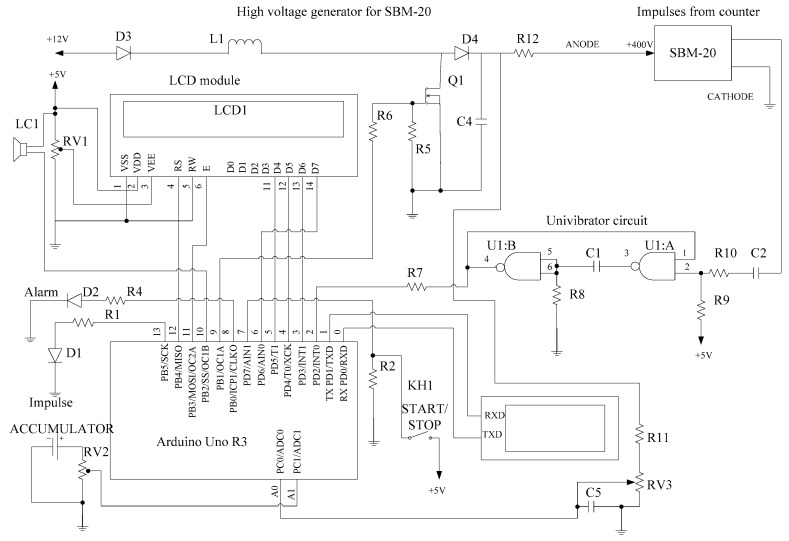
Circuit diagram of the Arduino microcontroller based system for radiation monitoring [15].

In Figure 6, the circuit of the microcontroller-based radiation monitoring system is shown. The system monitors the power of radiation, displays information and transmits the output data through the serial port to the PC.

As the radiation power increases more than the permissible norms (0.3 µSv/h), the sound (LS1 buzzer) and the light (the red LED “Alarm” blinks) alerts are switched on.

The indication of the intensity of the radiation impulses occurs with the blue LED1. In the LCD module the information about the radiation power in μSv/h and CPM (counts per minute) is continually displayed.

By pressing the key KH1 “START/STOP” the timer starts and additionally the obtained radiation dose during the period (μSv) is displayed on the LCD module.

For the correct operation of the system, additionally the accumulator (rechargeable battery) charge status and the supply voltage of the SBM-20 counter are measured (the ADC inputs of the Arduino Uno board A1, A0, respectively). The high voltage of the counter is generated with the pulse width modulator at the output 9 of the Arduino Uno board. It is controlled by the ADC input A0. The high voltage generator consists of the coil L1, transistor Q1, diode D4, resistors R6, R5, R11, RV3.

The short impulses from the SBM-20 counter arrive using the single-vibrator (univibrator) (the U1 chip, elements C1, C2, R10, R9, R8, R7) to the input INT0 of the Arduino Uno board for detection and analysis.

The developed hardware of the microcontroller-based radiation monitoring system has a low cost and the modular structure [15,22,23,24,25] that makes it possible to rapidly improve the system.

## 5. Features of Specialized Software Development for the Microcontroller-Based Background Radiation Monitoring System

The system software is written in Arduino IDE [26,27] with the maximum use of libraries of this environment and tools for downloading compiled code into the board. In particular, as an example, the software implementation of the algorithm for adjusting the anode voltage of the Geiger–Mueller radiation counter according to Figure 3 is shown in Appendix A.

The developed software has been tested [28]. It has a modular structure that enables the system to be upgraded quickly and efficiently [29]. In addition, the specialized software makes it possible to extend the functionality of the system when needed.

## 6. Simulation and Analysis of the Operation of the Microcontroller-Based System for Background Radiation Monitoring

**Figure 7:**
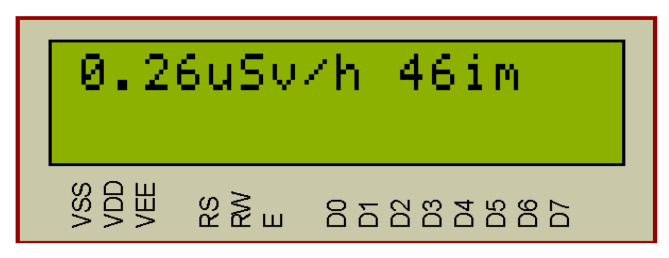
Simulation of the microcontroller-based system for background radiation monitoring (radiometer operation mode).

**Figure 8:**
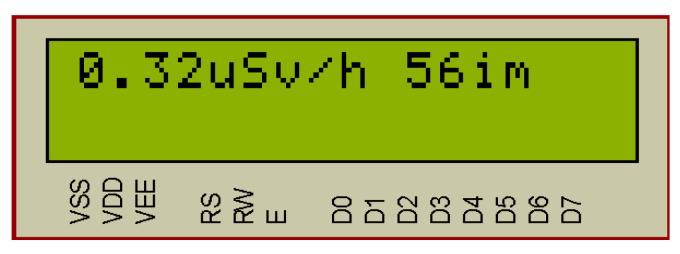
Simulation of the situation of exceeding the allowed radiation dose.

**Figure 9:**
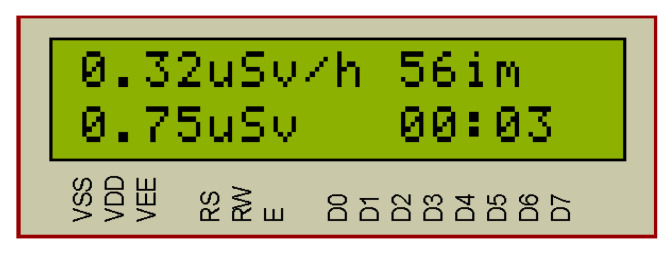
Simulation of the system operation in dosimeter mode.

The compiled program in Arduino Software (IDE) [26,27] for the Arduino Uno board (based on the ATmega328P microcontroller) is a hex file. The hex file is flashed into the microcontroller of the Arduino board. In the Figure 7, Figure 8 and Figure 9, the results of the operation simulation of the developed system are shown.

In the radiometer mode, Figure 7, the radiation power in μSv/h and the number of impulses per minute are displayed in the LCD module. The blue LED D1 “Impulse” blinks when the incoming impulse is detected. Simultaneously, the information is displayed in the LCD and sent to the PC via the serial interface (Table 1). Information update occurs every minute.

In Figure 8 the simulation results of the radiation dose exceeding are shown. The critical radiation dose is set during programming and is equal to 0.3 μSv/h. When the value of this dose exceeds, the red “Alarm” LED and alarm sound (LS1) are switched on. If the radiation is within the set tolerances, then the “Alarm” signals are off.

In Figure 9 the results of the system simulation in dosimeter mode are shown. When the KH1 button (“Start/Stop”) is pressed, the timer switches on and the system displays on the LCD screen the value of the accumulated radiation dose during the timer operation. The maximum timer value is 24 h. then the account is executed from scratch. The timer displays minutes and hours. The accumulated radiation dose in μSv. Pressing the KH1 key again stops the dosimeter mode and resets the LCD.

The obtained results of the device operation testing can be confirmed by the following data from the official technical documentation on the Geiger–Mueller counter SBM-20 [29,30,31,32,33], its radium calibrated sensitivity (Ra-226) is 29 cps/mR/h, cobalt calibrated sensitivity (Co-60) 22 cps/mR/h. The formula for converting the number of impulses per minute into the accumulated radiation dose in μSv/h is as follows:

[cpm]CF = [μSv/h].(3)

The conversion factor (CF) (conversion factor) from cpm into µSv/h for SBM-20:

29 cps/mR/h − 29 × 60 cpm/mR/h = 1740/8.77 (1 mR/h ~= 8.77 μSv/h)~ = 198 cpm/μSv/h, 1 cpm ~ 1/198 = 0.005040 µSv/h.

Therefore, CF = 0.00504 (Ra-226).

22 cps/mR/h – 22 × 60 cpm/mR/h = 1320/8.77 ~= 150 cpm/μSv/h, 1 cpm ~ 1/150 = 0.006644 µSv/h.

Thus, CF = 0.00664 (Co-60).

For SBM-20 the conversion factors are: CF = 0.00504 (Ra-226) and CF = 0.00664 (Co-60). In the software of the developed system, the average value of the coefficient CF = 0.0058 is taken, therefore, 56 imp. corresponds to a radiation dose of 0.32 μSv/h (μSv/h).

Consequently, the obtained results of the simulation and testing of the developed device and their coincidence with the theoretical calculations allow us to assert that the implemented system operates correctly.

## 7. Discussion of the Obtained Results

The obtained results of testing the developed physical model of the system in different modes coincide with the theoretical grounding and allow us to assert that the device is designed and implemented correctly and performs all previously defined functions. The system makes it possible to measure the equivalent dose of radiation, and the accumulated dose for a given period, outputs the received information on the serial port to the PC, and also gives warning signals in excess of the equivalent dose rate. Output information is displayed on the liquid crystal display.

A distinctive feature of the developed radiation background monitoring system is its multifunctionality, since inexpensive components are used, and the functionality of the device can be changed by improving the software of the microcontroller. In addition, the feature of the developed system is the ability to operate autonomously on accumulators and permanently from the 220 V network.

The limits of the developed system regarding the value and accuracy of the dose of radiation are determined by the parameters of the Geiger–Muller counter [21,29,30,31,32,33], namely, the upper limit of the radiation dose range can be adjusted using the built-in software.

However, it should be noted that the technical solution using the Arduino Uno platform is associated with a number of problems for the mobile version of the radiation background monitoring system. Accordingly, for such an option one must use industrial microcontrollers, which, in turn, significantly increases the cost of the system.

The further improvement of the developed device is possible by means of using the STM family of microcontrollers [34,35,36], which will increase the reliability [24,25,28]. In addition, the use of microcontrollers enables one to increase the functionality of the background radiation monitoring system.

## 8. Conclusions

In this paper, we have developed a background radiation monitoring system using the Arduino Uno board (based on the ATmega328P microcontroller) and the SBM-20 Geiger counter tube. The block diagram and operation algorithm for such a system have been composed. In addition, to research the operation dynamics of the designed system, we have built a model based on the theory of Petri nets. Next, we have built a low-cost physical model of the system. Appropriate software has also been developed. It allows us to implement wide-range functionality. The developed microcontroller-based system for background radiation monitoring can operate in both radiometer and dosimeter mode. The microcontroller-based radiation monitoring system measures the intensity of radiation in the radiometer mode. In the dosimeter mode, the microcontroller device determines an accumulated radiation dose over a period of time (max. 24 h), as well as gives alarm signals when the intensity of radiation is exceeded. The system can operate autonomously on accumulators and in stationary situations from the 220 V electricity network. The high reliability of the microcontroller-based system for background radiation monitoring is ensured by the rapid system response to the emergency situations: excess of the allowable intensity of equivalent radiation dose and the accumulator charge control. To conclude, our radiation monitoring system can be used in stationary premises as well as in mobile measurements.

## Figures and Tables

**Table 1:** Output of radiation dose in uSv/h and counts per minute.

cpm	uSv/h
0	0.00
46	0.26
42	0.24
45	0.26
43	0.25

## Appendix A. Software Implementation of the Algorithm for Adjusting the Anode Voltage of the Geiger–Mueller Counter

*void HV(void) // function of generation and control of the counter anode voltage*

*{*

*anod = analogRead(0); // analog Read – pin A0 – anode voltage*

*k = 127; // duty = 50%*

*a = (anod * 100)/1024; // anode voltage * 10*

*a = a * 10;*

*if (a < UHV)*

*{k = k + krok; // duty voltage increasing*

*if (k > 250)*

*{k = 250;};*

*}*

*else*

*{k = k − krok; // duty voltage decreasing*

*if (k < 0)*

*{k = 5;};*

*};*

*analogWrite(PWM, k); // PWM – anode voltage setting*

*}*
